# Leishmaniose généralisée de l'Ancien monde : Premier cas marocain chez un adulte immunocompétent ?

**DOI:** 10.48327/mtsi.2021.90

**Published:** 2021-11-05

**Authors:** Soumiya CHIHEB, Zineb TAZI SAOUD, Imane EL IDRISSI SAIK, Dounia DARIF, Fouzia HALI, Fatima Zahra El FATOIKI, Hayat Skali DAHBI, Ayyoub KIHEL, Ikram HAMMI, Maha SOUSSI ABDELLAOUI, Myriam RIYAD

**Affiliations:** 1 Service de dermatologie-vénérologie, Centre hospitalier Ibn Rochd, Casablanca, Maroc; 2 Laboratoire de pathologie cellulaire et moléculaire, Equipe: immunopathologie des maladies infectieuses et de système, Faculté de médecine et de pharmacie, Université Hassan II de Casablanca, Maroc; 3 Laboratoire biologie et santé, Faculté des sciences Ain Chock, Université Hassan II de Casablanca, Maroc; 4 Laboratoire de parasitologie-mycologie, Centre hospitalier Ibn Rochd, Casablanca, Maroc

**Keywords:** Leishmaniose généralisée, Leishmaniose viscérale, *Leishmania infantum*, Sujet immunocompétent, Hôpital, Casablanca, Maroc, Maghreb, Afrique du Nord, Generalised leishmaniasis, Visceral leishmaniasis, Leishmania infantum, Immunocompetent, Hospital, Casablanca, Morocco, Maghreb, Northern Africa

## Abstract

**Introduction:**

La leishmaniose dermique post kala-azar (LDPK) est un syndrome cutané observé après un traitement de leishmaniose viscérale (LV). Nous décrivons un cas probable chez un adulte immunocompétent.

**Observation:**

Il s'agit d'un homme âgé de 36 ans, originaire du sud du Maroc, avec antécédent de LV deux ans auparavant, traitée par antimoniate de méglumine et amphotéricine B avec une bonne évolution, hospitalisé en dermatologie pour un placard papulo-nodulaire érythémateux de la face. Le tableau dermatologique remontait à 6 mois avec une atteinte inaugurale de la muqueuse buccale. L'examen de la muqueuse buccale retrouvait une ulcération du tiers postérieur de la langue et un aspect papillomateux du voile du palais. La biopsie et le frottis cutanés retrouvaient des amastigotes de *Leishmania.* La PCR ITS1 était positive (genre *Leishmania).* Le diagnostic de LDPK était évoqué. Le patient a bénéficié d'injections intra-musculaires d'antimoniate de méglumine avec une bonne évolution.

**Conclusion:**

À notre connaissance, il s'agit du premier cas de leishmaniose généralisée évoquant une LDPK décrit au Maroc chez un adulte immunocompétent.

## Abstract (en)

**Background:**

Post-kala-azar dermal *Leishmaniasis* (PKDL) is a rare skin syndrome observed after treatment of visceral *Leishmaniasis* (VL) with pentavalent antimonial organic salts, never described in Morocco before. Here we report a case in an immunocompetent adult.

**Case:**

A 36-year-old-man from Tata in southern Morocco, with a history of visceral *Leishmaniasis* 2 years before and treated with meglumine antimoniate and amphotericin B with good clinical course, was hospitalized in dermatology for an erythematous papulo-nodular closet of the face. Six months ago, he presented oral mucosa involvement, then 3 months later, cutaneous lesions appeared on the face. The dermatological examination revealed a papulo-nodular erythematous closet extending to the nose and both cheeks, crusty and lupoid lesions on the forehead, around the eyes and chin, associated with an ulcerative and painless lesion on the heeL. The examination of the oral mucosa revealed an ulceration of the posterior third of the tongue and a papillomatous aspect of the soft palate. The skin biopsy and smear found some amastigote forms of *Leishmania* bodies. ITS1 PCR was positive (genus *Leishmania).* The HIV serology was negative. The diagnosis of PKDL was then evoked. The patient received intra-muscular injections of meglumine antimoniate with good progress.

**Conclusion:**

To our knowledge, this is the first case of generalised leishmaniasis suggesting PKDL reported in a Moroccan immunocompetent adult.

## Introduction

La leishmaniose dermique post kala-azar (LDPK) est un syndrome cutané rare observé après un traitement de leishmaniose viscérale (LV) par les sels organiques pentavalents d'antimoine et/ou amphotéricine B [13]. Initialement décrite au Soudan et en Inde, elle a été rattachée au kala-azar dû à *L. donovani,* mais quelques cas associés à la LV due à *L. infantum* ont été décrits dans les régions endémiques notamment chez des patients infectés par le virus de l'immunodéficience humaine (VIH) [3, 6, 17].

Au Maroc, la LV zoonotique due à *L. infantum* est endémique depuis plusieurs décennies. Après avoir longtemps été limitée au foyer de Taounate au nord du pays, elle s'est étendue dans le Nord et s'est même propagée hors de ses territoires historiques [12]. Par ailleurs, la LC est due à trois espèces: *L. major, L. tropica* et *L. infantum.* Jusqu'aux années 1990, la LC zoonotique due à *L. major* constituait la forme prédominante qui sévissait dans les provinces arides du sud du pays. Actuellement, la LC due à *L. tropica* considérée comme anthroponotique est caractérisée par la plus large distribution géographique [1]. En revanche, *L. infantum* est responsable de cas sporadiques dans le Nord, et notamment dans le Rif central [2]. Nous décrivons ici une observation clinique d'un patient marocain dont la symptomatologie et les antécédents évoquent une LDPK.

## Observation

Un patient de sexe masculin, âgé de 36 ans, originaire de Tata au sud du Maroc, consultait pour un placard papulo-nodulaire érythémateux de la face évoluant depuis 3 mois. Dans ses antécédents, on notait une hospitalisation deux ans auparavant au service des maladies infectieuses du CHU Ibn Rochd de Casablanca pour un syndrome d'insuffisance médullaire révélant une LV. Le patient avait été traité par antimoniate de méglumine (1,5 g/j) et amphotéricine B (100 mg tous les deux jours) durant 1 mois avec une bonne évolution.

**Figure 1:**
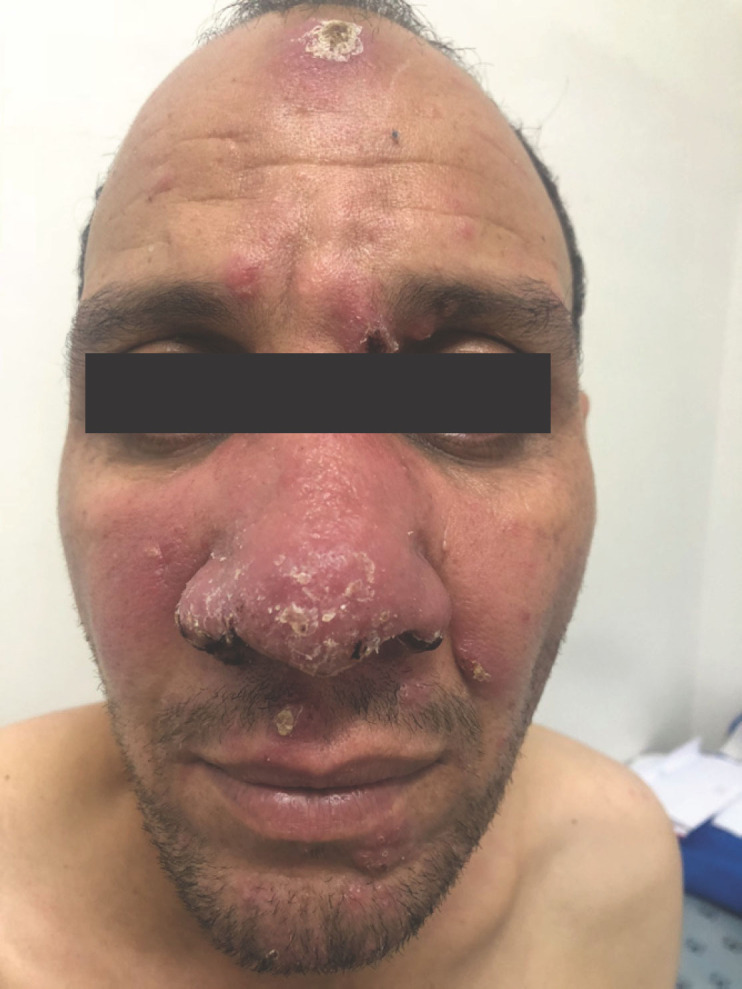
Placard facial papulo-nodulaire associé à des lésions croûteuses et lupoïdes du front Papulo-nodular facial plaque associated with crusted and lupoid lesions of the forehead

**Figure 2:**
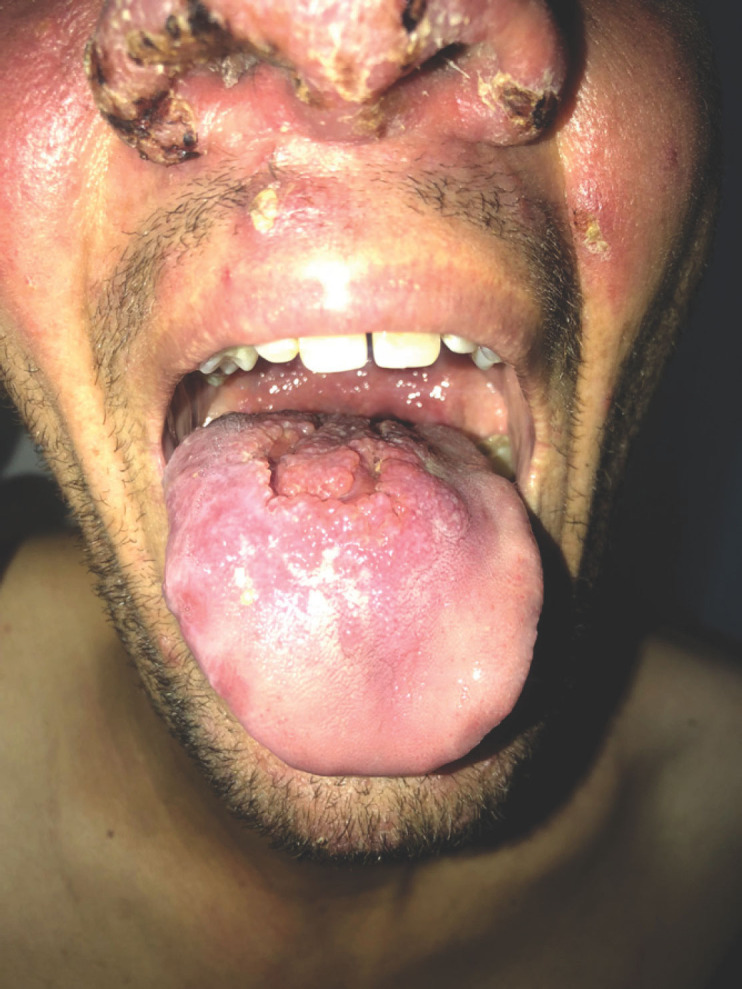
Ulcération circonférentielle du tiers postérieur de la langue Circumferential ulceration of the posterior third of the tongue

**Figure 3:**
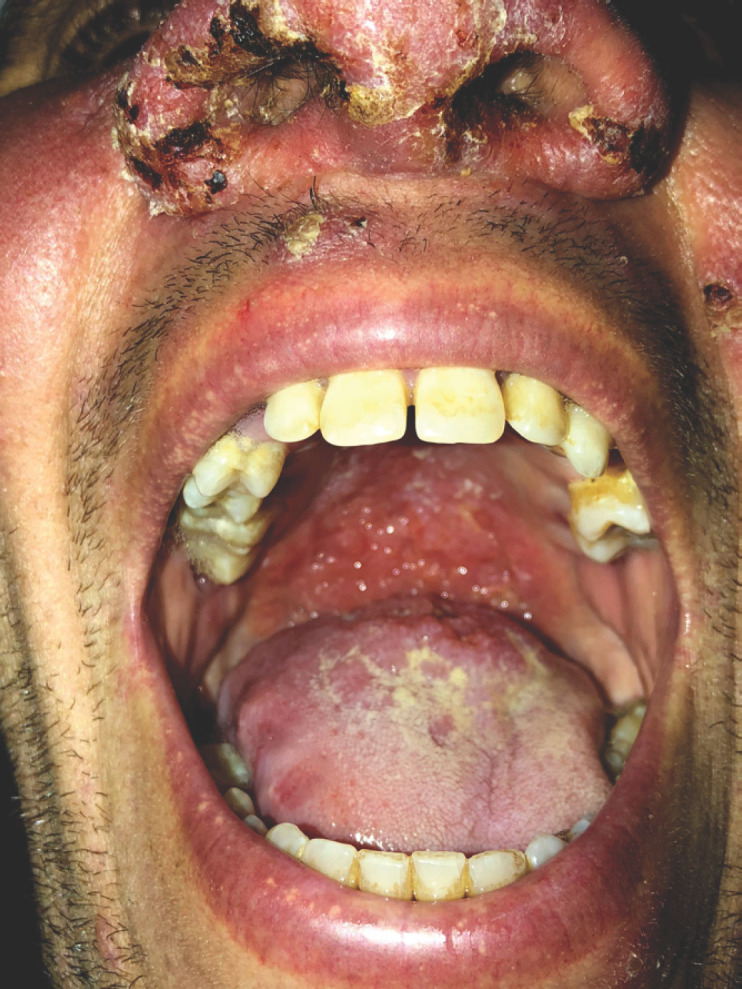
Aspect papillomateux du voile du palais Papillomatous appearance of the soft palate

Le tableau dermatologique remontait à 6 mois et se manifestait par une atteinte inaugurale de la muqueuse buccale entraînant une gêne à l'alimentation, puis par l'apparition trois mois plus tard de lésions cutanées de la face. Son examen clinique retrouvait un placard facial, érythémateux, papulo-nodulaire prenant le nez et les deux joues, des lésions croûteuses et lupoïdes du front (Fig. 1), du contour des yeux et du menton, associées à une lésion ulcéro-croûteuse et indolore du talon. L'examen de la muqueuse buccale retrouvait une ulcération circonférentielle du tiers postérieur de la langue (Fig. 2) et un aspect papillomateux du voile du palais (Fig. 3).

L'hémogramme retrouvait une leucopénie avec une lymphopénie à 860/μl et une thrombopénie à 103 000/µL. Le frottis cutané à la recherche d'amastigotes de *Leishmania* sp était positif. La biopsie cutanée objectivait un granulome avec la mise en évidence de formes amastigotes de *Leishmania* sp. La PCR ITS1 réalisée sur une lésion évolutive de la face était positive (genre *Leishmania),* mais la RFLP-HaeIII n'a pas permis d'identifier l'espèce (restriction négative). La sérologie (ELISA IgG *Leishmania infantum* DRG^**®**^) mettait en évidence la présence d'Ac *anti-Leishmania infantum* à un titre de 13 DRG Units versus 25,57 DRG Units au cours de sa première hospitalisation deux ans auparavant, avec confirmation du sérodiagnostic par mise en évidence de la bande de 15-18 kDa par Western Blot LDBIO-Diagnostics. Le myélogramme retrouvait quelques corps de Leishman.

Devant cette présentation inhabituelle, une sérologie VIH a été réalisée et s'est révélée négative. Le dosage des populations lymphocytaires B, T et NK par cytométrie en flux ne retrouvait pas de déficit, mais plutôt un aspect granuleux des lymphocytes et un excès en lymphocytes NK témoignant d'un processus infectieux actif. Le dosage des immunoglobulines A, G et M par turbidimétrie et des immunoglobulines E par technique immuno-enzymatique était normal.

**Figure 4:**
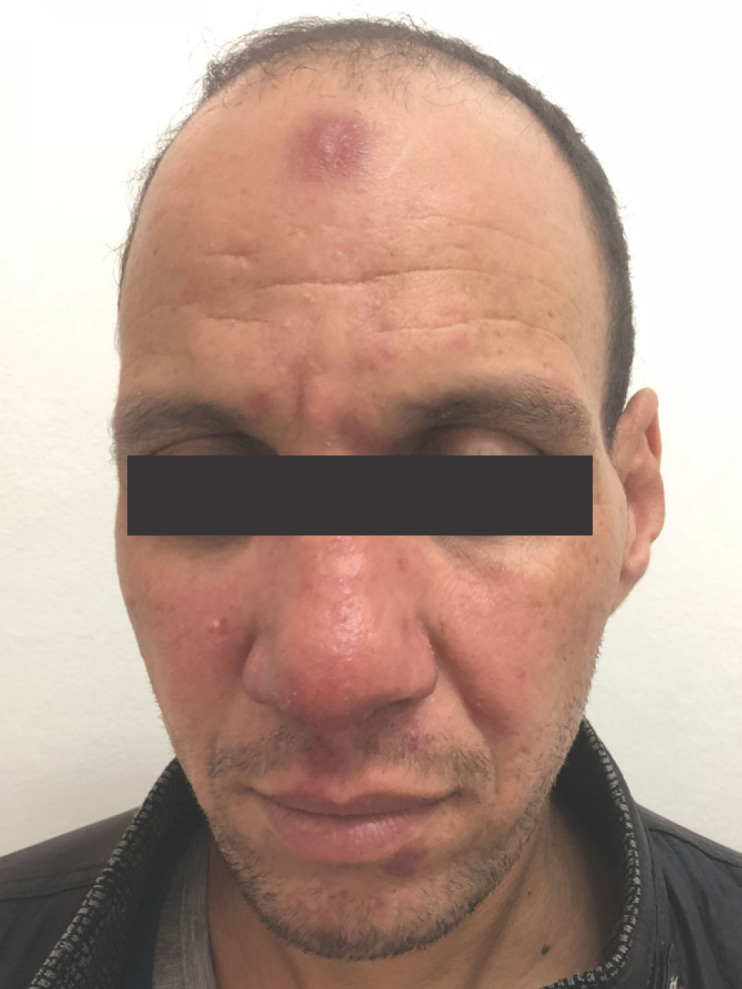
Désinfiltration des lésions cutanées Disinfiltration of skin lesions

**Figure 5:**
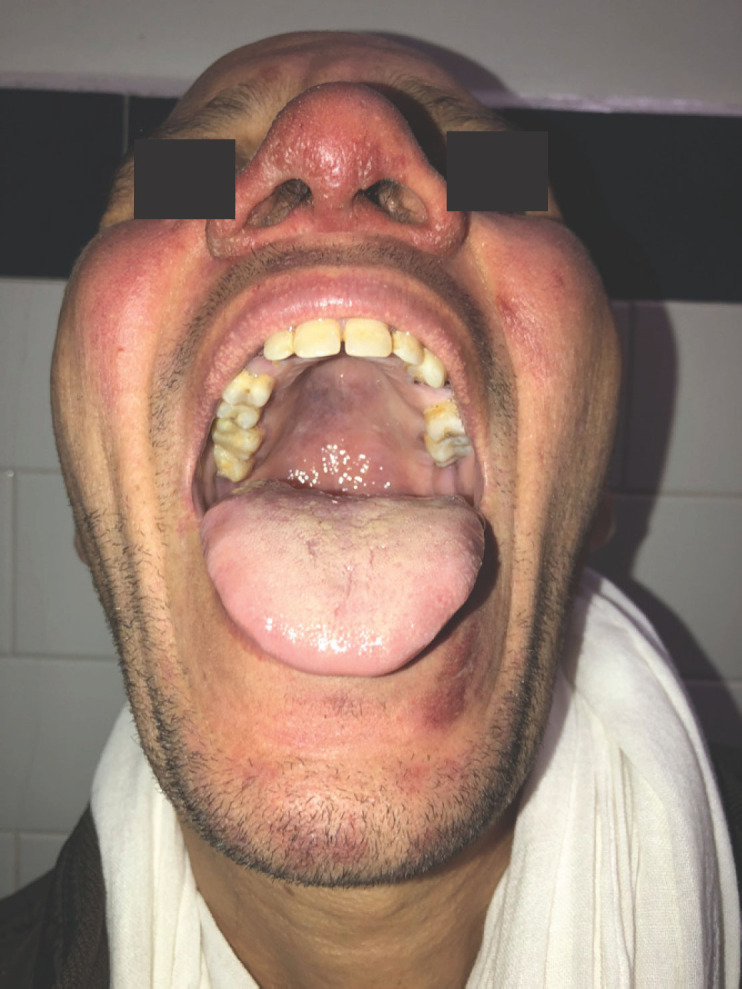
Cicatrisation des lésions muqueuses Healing of mucosal lesions

Ainsi, le diagnostic de LDPK a été évoqué devant les antécédents de LV traitée deux ans auparavant, la sémiologie des lésions cutanées, la présence d'amastigotes de leishmanies sur les examens parasitologiques et histologiques, appuyés par une PCR ITS1 positive. La sérologie VIH négative et le bilan immunologique ne retrouvaient pas de déficit immunitaire sous-jacent. Le malade était traité par des injections intra-musculaires d'antimoniate de méglumine (Glucantime^**®**^) à la dose de 20 mg/kg/j pendant 21 jours, avec une bonne tolérance clinique et biologique. L’évolution était marquée par la désinfiltration et la cicatrisation de toutes les lésions cutanées (Fig. 4) et muqueuses (Fig. 5) et la négativation du myélogramme. La sérologie leishmaniose, six semaines plus tard, restait positive au même taux. Notre recul actuel est d'un an. Des contrôles clinique et sérologique sont prévus, ainsi qu'un bilan de déficit immunitaire en dehors de tout épisode infectieux.

## Discussion

La particularité de notre observation réside dans la rareté de ce tableau de leishmaniose généralisée évoquant une leishmaniose cutanée post kala azar avec sa participation muqueuse exceptionnelle et sévère, et sa survenue en l'absence d'immunodépression sous-jacente.

La LDPK a été décrite pour la première fois par Brahmchari et al en Inde en 1922 [5]. Depuis, elle a fait l'objet de publications majoritairement indiennes et soudanaises où elle a été associée à *L. donovani* [20, 22]. Elle est plus fréquente chez les nourrissons, les malnutris, les immunodéprimés (VIH+), et chez les patients ayant reçu un traitement incomplet de LV [13].

Sur le plan clinique, les lésions cutanées débutent généralement autour de la bouche, avec extension secondaire au reste de la face, et plus rarement aux membres et au tronc. En Inde, elles apparaissent chez 20 % des patients traités pour kala-azar, 2 à 10 ans après le traitement. Elles se présentent le plus souvent sous forme de macules hypochromiques, posant un problème de diagnostic différentiel avec une lèpre ou un vitiligo [22]. Au Soudan, les lésions cutanées surviennent chez 50 à 60 % des patients traités pour kala-azar, moins de 12 mois après le traitement. Il s'agit le plus souvent de lésions papulo-nodulaires érythémateuses [20]. Notre patient a présenté le même type de lésions et le délai entre la LV et les lésions cutanées était de 18 mois. L’érythème des joues rapporté dans notre observation a été identifié comme étant un signe précoce [6].

L’étiopathogénie de la LDPK demeurant mal élucidée, les auteurs s'accordent sur le fait que durant le traitement d'une LV, les parasites se réfugieraient au niveau du derme et qu'un traitement incomplet induirait la réactivation des parasites latents [22] Cependant, quelques cas de LDPK ont été décrits chez des patients asymptomatiques infectés par *L. donovani,* sans antécédent de LV [7].

L'implication muqueuse de la LDPK a rarement été observée dans l'Ancien Monde soulignant l'originalité de cette observation [16, 18]. Des atteintes muqueuses ont été rapportées au Moyen-Orient et sur le pourtour méditerranéen chez des malades souffrant d'une LV concomitante, d'une LDPK, et chez les personnes âgées ou immunodéprimées. Or, notre patient était séronégatif pour le VIH et immunocompétent. Son atteinte muqueuse avait précédé de trois mois les lésions cutanées, sans lesquelles le diagnostic de leishmaniose n'aurait peut-être pas été évoqué. Le diagnostic positif de la LDPK repose sur un faisceau d'arguments anamnestiques, parasitologiques, immunologiques, histologiques et sérologiques. L'anamnèse doit noter l'existence d'un antécédent de LV traitée. Les lésions muqueuses et papulo-nodulaires seraient plus souvent positives par rapport aux lésions maculeuses [19]. Les variantes maculaires et hypopigmentées correspondent à une réaction granulomateuse isolée et pauciparasitaire. Les formes érythémateuses et nodulaires, plus courantes, font apparaître une infiltration histiocytaire, un œdème, une prolifération des capillaires et de nombreux parasites [15]. Les techniques de biologie moléculaire confirmeraient le diagnostic. Il faut noter qu'un taux résiduel de parasites après une cure de LV serait en faveur d'un risque plus élevé de développer une LDPK [19]. Or le myélogramme de notre patient s'est révélé positif après deux ans. Par ailleurs, les examens sérologiques (test d'agglutination directe, ELISA) ne sont pas d'un grand intérêt puisque les anticorps peuvent persister jusqu’à 2 ans après une LV traitée. Cependant, ils sont utiles pour le diagnostic différentiel ou si les antécédents de LV sont incertains [21]. Chez notre patient, la persistance des anticorps même après traitement des lésions serait en faveur d'une cicatrice sérologique. Néanmoins, même si la sérologie est en faveur d'une infection à *L. infantum,* et étant donné que l'espèce n'a pu être identifiée, nous ne pouvons pas éliminer une potentielle infection concomitante à *L. major* ou *L. tropica.*

Dans le monde, les parasites isolés des patients atteints de LDPK sont identiques au plan biochimique et moléculaire aux souches de *L. donovani* isolées à partir des patients atteints de kala-azar [9]. Dans les régions endémiques de *L. infantum,* quelques cas ont été décrits, surtout chez les patients VIH [3, 13, 17]. La LV zoonotique due à *L. infantum* dans la région méditerranéenne est classiquement une pathologie du jeune enfant en zone rurale et les formes adultes de l'immunocompétent sont peu fréquentes. Néanmoins, chez notre patient, il est fort probable qu'il s'agisse de *L. infantum* au vu de ses antécédents et de la sérologie anti-*Leishmania infantum* positive. La virulence du parasite serait due à la migration et à l'introduction récente de *L. infantum* au sud du Maroc, favorisée par des changements climatiques et environnementaux et se traduisant cliniquement par des formes graves de leishmaniose cutanée [4, 10].

Une étude a montré que 16 % des patients présentant une LDPK (37 sur 233 patients) présentaient une localisation lymphatique (leishmanies mises en évidence sur une biopsie ganglionnaire) et médullaire (leishmanies mises en évidence sur le myélogramme comme chez notre patient) [20].

Le traitement de LDPK n'est pas codifié. Devant la guérison spontanée de la majorité des malades en Afrique centrale, le traitement n'est pas systématique. Seuls sont traités les sujets gravement atteints, ceux qui présentent des lésions défigurantes, ceux dont les lésions persistent pendant plus de 6 mois, ceux qui souffrent d'une uvéite concomitante [11, 16] ou encore les jeunes enfants porteurs de lésions buccales qui les empêchent de s'alimenter [20]. En Inde, le traitement est systématique en raison de la persistance des lésions pendant une longue durée faisant du patient un réservoir chronique participant à la transmission du kala-azar, considéré comme une anthropozoonose [8, 14, 22]. Chez notre patient, nous avons eu recours à l'antimoniate de méglumine (Glucantime^**®**^) en injections intra-musculaires pendant une durée de trois semaines; la guérison était retenue sur la régression clinique de toutes les lésions et la négativation des examens parasitologiques.

## Conclusion

À notre connaissance, ceci est la première description d'une leishmaniose généralisée évoquant une LDPK chez un adulte immunocompétent au Maroc. Il illustre la nécessité de suivre les patients traités. Non traités, les patients abritent le parasite dans les lésions cutanées pendant des années et pourraient constituer un réservoir du parasite. Ainsi, ces patients infectés par *L. infantum,* dont le cycle naturel est zoonotique, pourraient contribuer à créer un cycle anthroponotique qui pourrait avoir des répercussions sur l’épidémiologie de la LV due à *L. infantum* dans notre région et sur les mesures de prévention.

## Liens d'intérêts

Les auteurs déclarent ne pas avoir de liens d'intérêt.

## Contribution des auteurs

Soumiya CHIHEB: analyse, rédaction, révision et correction du manuscrit. Zineb TAZI SAOUD: recueil des données, analyse, rédaction, et correction du manuscrit. Imane EL IDRISSI SAIK: recueil des données, génotypage, rédaction du manuscrit. Dounia DARIF: recueil des données, génotypage. Fouzia HALI: révision du manuscrit. Fatima Zahra El FATOIKI: révision du manuscrit. Hayat Skali DAHBI: révision du manuscrit. Ayyoub KIHEL: génotypage. Ikram HAMMI: génotypage. Maha Soussi ABDELLAOUI: diagnostic parasitologique direct et sérologique, interprétation et validation des résultats. Myriam RIYAD: analyse, rédaction, révision et correction du manuscrit.

## References

[1] Aoun K, Bouratbine A (2014). Cutaneous *Leishmania*sis in North Africa: a review. Parasite.

[2] Asmae H, Fatima A, Hajiba F, Mbarek K, Khadija B, Mohamed R, Faiza S (2014). Coexistence of *Leishmania* tropica and *Leishmania infantum* in Sefrou province, Morocco. Acta Trop.

[3] Badirzadeh A, Mohebali M, Ghasemian M, Amini H, Zarei Z, Akhoundi B, Hajjaran H, Emdadi D, Molaei S, Kusha A, Alizadeh S (2013). Cutaneous and post kala-azar dermal *Leishmania*sis caused by *Leishmania infantum* in endemic areas of visceral *Leishmania*sis, northwestern Iran 2002-2011: a case series. Pathog Glob Health.

[4] Baghad B, Razanapinaritra R, Maksouri H, El Bouri H, Outlioua A, Fellah H, Lemrani M, Akarid K, Martin-Sanchez J, Chiheb S, Riyad M (2020). Possible introduction of *Leishmania* tropica to urban areas determined by epidemiological and clinical profiles of patients with cutaneous *Leishmania*sis in Casablanca (Morocco). Parasite Epidemiol ControL.

[5] Brahmachari UN (1922). A New Form of Cutaneous *Leishmania*sis-Dermal Leishmanoid. Ind Med Gaz.

[6] Catorze G, Alberto J, Afonso A, Vieira R, Cortes S, Campino L (2006). Co-infection *Leishmania infantum*/VIH: lésions cutanées après traitement d'une leishmaniose viscérale. Ann Dermatol VenereoL.

[7] Das VN, Pandey RN, Siddiqui NA, Chapman LA, Kumar V, Pandey K, Matlashewski G, Das P (2016). Longitudinal Study of Transmission in Households with Visceral *Leishmania*sis, Asymptomatic Infections and PKDL in Highly Endemic Villages in Bihar, India. PLoS Negl Trop Dis.

[8] Duthie MS, Goto Y, Ghosh P, Mondal D (2019). Impact of sequelae of visceral *Leishmania****sis*** and their contribution to ongoing transmission of *Leishmania* donovani. Pathog Dis.

[9] Ganguly S, Das NK, Barbhuiya JN, Chatterjee M (2010). Post-kala-azar dermal *Leishmania****sis–an*** overview. Int J DermatoL.

[10] Chiheb S, Hamdani A, Riyad M, Bichichi M, Hamdani S, Krimech A (1997). Cutaneous *Leishmania*sis: an emerging epidemic focus of *Leishmania* tropica in north Morocco. Trans R Soc Trop Med Hyg.

[11] Khalil EA, Musa AM, Younis BM, Elfaki ME, Zijlstra EE, Elhassan AM (2011). Blindness following visceral *Leishmania*sis: a neglected post-kala-azar complication. Trop Doct.

[12] Kholoud K, Bounoua L, Sereno D, El Hidan M, Messouli M (2020). Emerging and Re-Emerging *Leishmania*ses in the Mediterranean Area: What Can Be Learned from a Retrospective Review Analysis of the Situation in Morocco during 1990 to 2010?. Microorganisms.

[13] Mokni M (2019). Leishmanioses cutanées. Ann Dermatol VenereoL.

[14] Mondal D, Bern C, Ghosh D, Rashid M, Molina R, Chowdhury R, Nath R, Ghosh P, Chapman LAC, Alim A, Bilbe G, Alvar J (2019). Quantifying the Infectiousness of Post-Kala-Azar Dermal *Leishmania*sis Toward Sand Flies. Clin Infect Dis.

[15] Ramesh V, Singh R, Salotra P (2007). Short communication: post-kala-azar dermal *Leishmania*sis–an appraisaL. Trop Med Int Health.

[16] Ramos A, Cruz I, Muñez E, Salas C, Fernández A, Alvarez-Espejo T (2008). Post-kala-azar dermal *Leishmania*sis and uveitis in an HIV-positive patient. Infection.

[17] Ridolfo AL, Gervasoni C, Antinori S, Pizzuto M, Santambrogio S, Trabattoni D, Clerici M, Galli M (2000). Post-kala-azar dermal *Leishmania*sis during highly active antiretroviral therapy in an AIDS patient infected with *Leishmania infantum*. J Infect.

[18] Shirian S, Oryan A, Hatam GR, Daneshbod Y (2013). Three *Leishmania****/L.*** species - *L. infantum, L. major, L. tropica* - as causative agents of mucosal *Leishmania*sis in Iran. Pathog Glob Health.

[19] Verma S, Bhandari V, Avishek K, Ramesh V, Salotra P (2013). Reliable diagnosis of post-kala-azar dermal *Leishmania*sis (PKDL) using slit aspirate specimen to avoid invasive sampling procedures. Trop Med Int Health.

[20] Zijlstra EE, Khalil EA, Kager PA, El-Hassan AM (2000). Post-kala-azar dermal *Leishmania*sis in the Sudan: clinical presentation and differential diagnosis. Br J DermatoL.

[21] Zijlstra EE (2019). Biomarkers in Post-kala-azar Dermal *Leishmania*sis. Front Cell Infect MicrobioL.

[22] Zijlstra EE, Alves F, Rijal S, Arana B, Alvar J (2017). Post-kala-azar dermal *Leishmania*sis in the Indian subcontinent: A threat to the South-East Asia Region Kala-azar Elimination Programme. PLoS Negl Trop Dis.

